# Viral Hepatitis in Nepal: Past, Present, and Future

**DOI:** 10.5005/jp-journals-10018-1169

**Published:** 2016-07-09

**Authors:** Ananta Shrestha

**Affiliations:** Department of Hepatology, The Liver Clinic, Liver Foundation Nepal, Tripureshwor, Kathmandu, Nepal

**Keywords:** Hepatitis E, Kathmandu valley, Kinetic of hepatitis, Viral hepatitis.

## Abstract

Over the last four decades, the diagnosis and therapy of viral hepatitis has evolved substantially all over the world. Although Nepal is a developing nation, it has been keeping pace with these developments in terms of the implementation of diagnostic and therapeutic strategies. However, because of a lack of infrastructure and logistics, Nepal still depends on its neighboring countries for molecular diagnostics in viral hepatitis B and C. Though most of the recent antivirals for hepatitis B and directly acting antivirals for hepatitis C are available at a highly subsidized cost, affordability still remains an issue. A lack of comprehensive national program for hepatitis B and C has been a major barrier for access to health care in patients with chronic viral hepatitis. Similarly, hepatitis E still remains an important public health issue with major epidemics at periodic intervals. Recent developments in understanding hepatitis E virus have unveiled important aspects of the virus, particularly with regard to why these epidemics occur. In this manuscript we try to elaborate the evolution in understanding, diagnosing, and treating viral hepatitis in Nepal.

**How to cite this article:**

Shrestha A. Viral Hepatitis in Nepal: Past, Present, and Future. Euroasian J Hepato-Gastroenterol 2016;6(1):59-61.

## INTRODUCTION

Nepal is a small South Asian country sandwiched between India and China with a population of 2.6 million. The geographical and ethnic diversity of this nation provides an opportunity to study the blend of unique epidemiology of viral hepatitis. Viral hepatitis epidemiology is a dynamic entity. It is subjected to change with socioeconomic development, evolution of sociocultural practices, community interventions implemented by national programs, and evolving understanding of hepatitis viruses.

There has been tremendous development in understanding viral hepatitis across the globe. While hepatitis A and B were the only known hepatotropic viruses and Non A Non B (NANB) hepatitis was used to designate all other infectious hepatitis three decades earlier, the discovery of hepatitis C and E has changed the understanding about chronic liver disease and acute hepatitis. Further, the development of molecular techniques, antiviral medications, and effective vaccines against some of these viruses has changed our ways to tackle with them.

## HEPATITIS B VIRUS

The first ever testing for hepatitis B surface antigen (HBsAg) in Nepalese subjects was done at the Yale University in sera collected during acute hepatitis epidemic of 1973. When HBsAg was found to be negative in these subjects, the epidemic was presumed and reported as hepatitis A epidemic.^1^ Commercial testing for HBsAg has been made available in Nepal since 1983. Subsequently, a nationwide study revealed the prevalence of hepatitis B to be 0.9% on an average (1.1% among males and 0.5% among females).^2^ Some ethnic groups were found to have high prevalence of hepatitis B virus (HBV) infections as compared to the general population. It accounts for nearly 3% of sporadic acute hepatitis cases, 47% of liver cirrhosis, and 69% of hepatocellular carcinoma.^3^ Vaccination against hepatitis B was incorporated in the Extended Program on Immunization (EPI) schedule in 2002 and is given in combination with DPT vaccine starting from the age of 6 weeks. Antiviral medications have been available since 2001 when Lamivudine was first introduced in the country. Subsequently, adefovir, tenofovir, and entecavir became commercially available.

It is estimated that nearly 260,000 individuals are chronically infected with HBV in the country and a majority of them are unaware of their infection. There are limited numbers of health care providers properly trained in the management of chronic hepatitis B infection. The majority of the burden is tackled by general physicians, and a standard protocol for workup, follow-up, and treatment is lacking. Nepal still lacks a facility for the estimation of HBV DNA viral load and is entirely dependent on Indian commercial laboratories. The need for exporting the samples to Indian cities for molecular testing often leads to sample degradation and inaccurate results.

Currently the country has a successfully running Anti-Retroviral Treatment (ART) program. However, a viral hepatitis program has not been prioritized and is still a far outcry. A well-designed hepatitis B control and treatment program with a comprehensive national guideline is felt highly necessary. Such programs should address strategies for population screening, monitoring, and treating chronic hepatitis B subjects in order to reduce HBV-related morbidity and mortality.

## HEPATITIS C VIRUS

Laboratory testing for hepatitis C virus (HCV) in Nepal started in 1991. It accounted for a small proportion of chronic hepatitis and cirrhosis patients among whom alcohol and HBV were excluded. In a general population survey, the seroprevalence of HCV was found to be 0.6% in the mid-1990s.^4^ However, it was seen to be very common among intravenous drug users where anti-HCV was found to be positive in nearly 95% cases.^5^ About 74% of seropositive individuals had HCV viremia. Similarly, anti-HCV prevalence was noted to be high among hemophiliacs who received plasma products for hemorrhagic episodes. HIV co-infection appears to be a significant problem among anti-HCV-positive intravenous drug users. Nearly 28% of anti-HCV-positive individuals were found to have HIV co-infection. As compared to India and Pakistan, iatrogenic HCV infection does not seem to be a major contributor for HCV infection.

Seroprevalence of HCV has, however, not changed over the last 20 years. In 2008, anti-HCV was found to be positive in 0.66% of 33,255 healthy blood donors.^6^ The estimated total burden of HCV infection is 130,000 all over Nepal.

In studies done between 1997 and 2006, HCV accounted for 3% of acute viral hepatitis, 10% of chronic liver disease, and 14% of hepatocellular carcinoma.^3^ On the contrary, it is the leading cause of morbidity and mortality among intravenous drug users. Hepatitis C virus genotype 3 is by far the most common genotype accounting for nearly 72% of all HCV infection followed by genotype 1, accounting for nearly 18%. Genotype 2 was found in 3% and genotypes 4 and 5 account for 1% each. As with HBV, molecular testing for HCV viral load and genotyping is not available commercially in Nepal.

There has been substantial evolution in the drug therapy of HCV over the last decade, and it applies similarly to Nepal as well. The availability of treatment for HCV has been at par with any other developed countries starting from standard interferon to pegylated interferon and directly acting antivirals. However, only a small fraction of infected population have been able to access these treatments mainly owing to a lack of awareness and unaffordability. Till 2014, 24 weeks of therapy with pegylated interferon and ribavirin cost about 5,000 U.S. dollars. Limited SVR rates, adverse effects, and costs were barriers for the treatment of HCV in Nepal. With the availability of DAAs, the cost of treatment has dropped down to nearly 1,000 to 2,500 U.S. dollars for a full course of therapy across different regimens.

Despite the availability of DAAs at highly subsidized cost, it is still beyond affordability in most of the infected individuals. A national strategy to tackle the existing burden of disease and prevent new infection is lacking. The national body of hepatologists is currently advocating about HCV burden and the need for national strategies to gain government interest. A comprehensive disease control and treatment program should be formulated in the near future. This should address screening for HCV in the general population and high-risk population, making molecular testing available in the country, further subsidizing medications, and developing follow-up registries. Preventing new infections among intravenous drug abusers by education, needle exchange programs, and mop-up treatment of currently infected individuals could contain the burden of HCV among intravenous drug users. Incorporating information about hazards of intravenous drug abuse and HCV infection in the school curriculum could be an effective way of preventing both HCV and HIV infections.

## HEPATITIS E VIRUS

Kathmandu valley, the capital of Nepal, is a highly endemic region for hepatitis E virus (HEV) infection. Five epidemics have occurred in Kathmandu, 1973, 1981 to 1982, 1987, 2006 to 2007, and recently in Biratnagar city in 2014.^7^ Hilly rural areas that constitute the major landscape of the country, however, appear to be spared. Hepatitis E is rare in these areas and anti-HEV IgG prevalence is very low. Sporadic hepatitis E is seen in between the epidemics and accounts for 15 to 50% of acute hepatitis cases.^8,9^ Epidemics occur in periodic fashion and the numbers of cases of hepatitis E also tend to fluctuate in sporadic acute hepatitis. In recent years, hepatitis A virus (HAV) has outnumbered HEV infection in acute viral hepatitis among adults.

The periodicity and fluctuation in HEV cases in Kathmandu was presumed to be due to a change in herd immunity of the population. Internal migration of nonimmune population from rural areas into the Kathmandu valley for education and employment is thought to bring this shift in herd immunity. Fecal contamination of drinking water supply due to breach in underground pipelines triggers an outbreak when herd immunity is low. However, this concept has been challenged recently. In 2014, anti-HEV IgG was detected in 8% of healthy blood donors in Kathmandu valley, indicating low herd immunity. During the pre-monsoon period, HEV viremia was detected in 1.5% of asymptomatic healthy donors, indicating active ongoing transmission. However, clinical cases of HEV hepatitis were negligible in the valley and an epidemic did not occur despite low herd immunity and ongoing HEV transmission. This indicated that low herd immunity and fecal contamination may not be sufficient enough for an epidemic to occur and virological factors may also be responsible. In fact, in 2014 an epidemic of HEV occurred in Biratnagar city, where HEV hepatitis was not a common disease. Isolates of HEV from this epidemic were different from previous isolates of HEV from Kathmandu and neighboring countries. These isolates, in fact, have now been proposed to be a novel subtype of HEV. Further, a unique mutation was detected in the ORF 1 region in gene encoding for RNA-dependent RNA polymerase (RdRP) that offered replication fitness, increased pathogenicity, and *in vitro* resistance to ribavirin. Therefore, current concepts of HEV epidemiology have been evolving in recent years and two important new aspects of HEV have been unveiled: first, the occurrence of asymptomatic HEV carrier, which can act as a dynamic human reservoir, and second, the potential role of HEV mutation in causation of HEV epidemics. Further investigations are under way, which will further clarify and help understand these two phenomena in days to come.

## CONCLUSION

Since the beginning of hepatology in Nepal almost four decades ago, there has been rapid development in the understanding and availability of treatment for viral hepatitis. As with this development, different areas of needs are being realized, which shall be addressed in future.
